# Timing of Allergenic Food Introduction in Infants, Saudi Arabia: A Cross-Sectional Study

**DOI:** 10.3390/nu18060930

**Published:** 2026-03-16

**Authors:** Imad Khojah, Reham Alsaud, Zayna Fatani, Abdulaziz Alotaibi, Hadeel Alharbi, Elaf Bahareth, Hala Fatani, Loie Goronfolah, Husni Rayes, Mohammad Binhussein, Ameera Bukhari, Mohammed A. Almatrafi, Eilaf Fallatah, Amer Khojah

**Affiliations:** 1 Department of Emergency Medicine, Faculty of Medicine, King Abdulaziz University, Jeddah 21589, Saudi Arabia; 2 Department of Emergency Medicine, King Abdulaziz University Hospital, King Abdulaziz University, Jeddah 22252, Saudi Arabia; 3 College of Medicine, Umm Al-Qura University, Makkah 21955, Saudi Arabia; 4 Translational Medical Sciences, Nottingham Digestive Diseases Centre, School of Medicine, University of Nottingham, Nottingham NG7 2UH, UK; 5 National Institute for Health and Care Research (NIHR), Nottingham Biomedical Research Centre, Nottingham University Hospitals NHS Trust, University of Nottingham, Nottingham NG7 2UH, UK; 6 Department of Pediatrics, College of Medicine, King Saud Bin Abdulaziz University for Health Sciences, Jeddah 22384, Saudi Arabia; 7 Department of Pediatrics, Makkah Maternity and Children Hospital, Makkah 24246, Saudi Arabia; 8 Department of Internal Medicine, College of Medicine, Umm Al-Qura University, Makkah 21955, Saudi Arabia; 9 College of Science, Taif University, Taif 21944, Saudi Arabia; 10 Department of Pediatrics, College of Medicine, Umm Al-Qura University, Makkah 21955, Saudi Arabia

**Keywords:** food allergy, Atopy, pediatric allergy, food introduction, maternal awareness, Saudi Arabia

## Abstract

Background: Food allergy (FA) is an increasing public health concern with significant implications for child health and quality of life. Early introduction of allergenic foods has been shown to reduce the risk of food allergy development; however, maternal awareness and adherence to these recommendations remain inconsistent. This study aimed to assess maternal awareness and practices regarding the timing of allergenic food introduction among mothers residing in Makkah, Saudi Arabia. Methods: A descriptive cross-sectional study was conducted between November 2023 and March 2024 involving parents of children aged younger than 48 monthsin the Makkah region. Data were collected via a self-administered electronic questionnaire distributed through social media platforms. Results: A total of 391 parents participated. Parent-reported food allergy was identified in 11.3% of children, while 14.6% had eczema. Early introduction (<12 months) was more common for egg (43.3%) and wheat (71.1%) compared to peanut (28.9%), tree nuts (30.9%), sesame (30.9%), and seafood (28.9%). A considerable proportion of children had not been introduced to key allergenic foods even after 36 months, particularly peanuts (45.3%) and sesame (42.2%). Children with eczema were significantly more likely to have early introduction of egg (*p* = 0.035), tree nuts (*p* = 0.046), and seafood (*p* = 0.031). Similarly, children with a family history of food allergy had higher early introduction rates of tree nuts (55.3% vs. 44.0%, *p* = 0.043) and seafood (62.3% vs. 49.1%, *p* = 0.019). Only 25.8% of mothers were aware that early introduction might prevent food allergies, and just 22% reported receiving professional advice to introduce allergenic foods early. Conclusions: Maternal awareness regarding the timely introduction of allergenic foods in Makkah remains limited, with delayed introduction persisting beyond 36 months for several high-risk allergens. These findings underscore the need for targeted educational interventions and improved counseling by healthcare providers.

## 1. Introduction

Food allergy (FA) is an immune-mediated hypersensitivity reaction to specific food and is a growing public health concern due to its impact on quality of life and the risk of life-threatening anaphylaxis [1,2]. Globally, the prevalence of FA varies by geography, dietary habits, and diagnostic criteria [3]. For example, the prevalence of food allergy among Korean infants has been reported at 5.3%, which is comparable to rates observed in the United Kingdom [4,5]. In the United States, approximately 7.6–8% of children are affected, with peanuts, milk, and eggs being the most common allergens [6,7]. In contrast, recent studies from Saudi Arabia report higher prevalence rates [8]. A national cross-sectional study estimated the prevalence of parent-reported FA in children at 15.2% [8]. Common allergens identified in Saudi pediatric populations include eggs, tree nuts, peanuts, milk, and sesame [8]. A similar study conducted in Taif reported a prevalence of 16.1% [9], underscoring the substantial burden of FA among Saudi pediatric populations.

The development of FA is influenced by a combination of genetic and environmental factors [10,11]. Children with atopic comorbidities such as eczema, or allergic rhinitis, as well as those with a first-degree relative with allergies, are at heightened risk for food sensitization and allergy development [12,13,14]. In recent years, a paradigm shift in FA prevention has occurred. Evidence from landmark studies, such as the LEAP (Learning Early About Peanut Allergy) trial and the EAT (Enquiring About Tolerance) study, has demonstrated that the early introduction of allergenic foods significantly reduces the risk of food sensitization and allergy development [12,15,16,17,18]. As a result, several organizations such as American Academy of Pediatrics (AAP), Asia Pacific Association of Pediatric Allergy, Respirology & Immunology (APAPARI), and European Academy of Allergy and Clinical Immunology (EAACI) have all issued recommendations supporting the early introduction of allergenic foods in infancy, especially in children at high risk for atopic disease [19,20,21,22].

Despite these recommendations, implementation of these guidelines in community settings remains suboptimal [23]. Maternal awareness of the benefits of early allergenic food introduction remains inconsistent globally and regionally [23]. In Saudi Arabia, previous studies have reported that approximately 62% of infants are introduced to solid foods before the age of 4 months [24], with some caregivers offering traditional foods such as dates mixed with water. This practice contradicts the World Health Organization (WHO) recommendation of exclusive breastfeeding for the first six months of life, which is also endorsed by the Saudi Ministry of Health [25,26,27]. Additionally, early introduction of honey before 1 year of age contrary to established guidelines remains a challenge in some rural communities [28]. Although infants may encounter allergenic proteins through breast milk, where cow’s milk, egg, wheat, and peanut proteins have been detected [29], their concentrations are variable, and the protective role of such exposure in preventing food allergy remains unclear. Several factors have been shown to influence early feeding practices, including paternal education level, cultural beliefs regarding infant feeding, and family history of food allergies [23,30,31,32].

Despite the high prevalence of FA in Saudi children and the global emphasis on early allergen introduction, limited data exist regarding maternal knowledge and practices related to allergenic food introduction in the region, particularly in western Saudi Arabia. Understanding these practices is essential to support public-health planning and improve early prevention strategies. Therefore, this study aims to assess maternal awareness of the timing of allergenic food introduction among mothers residing in Makkah, Saudi Arabia.

## 2. Materials and Methods

This descriptive cross-sectional study was conducted between November 2023 and March 2024 in the Makkah region of Saudi Arabia. Data were collected using a self-administered questionnaire adapted from a previously published study [33]. The survey link included a brief description of the study objectives and an electronic informed consent form. Twelve data collectors assisted with distributing the questionnaire across different areas of the Makkah region to enhance geographic coverage.

The questionnaire was distributed electronically via text messaging and various social media platforms, including X (formerly Twitter), WhatsApp, Instagram, and Facebook. The questionnaire underwent a pilot study involving 25 participants to assess clarity, language, and content validity. Feedback from pilot participants was independently reviewed by three board-certified allergists, and minor revisions were made to optimize clarity and applicability. For example, the instructions were refined to specify that responses should refer to the youngest child in households with multiple children.

Inclusion criteria included parents residing in the Makkah region with children aged younger than 48 months. Exclusion criteria included: (1) parents of children outside the specified age range, (2) healthcare professionals, (3) individuals who declined to participate, and (4) incomplete survey responses.

Sample size was calculated using Epi Info software version 2.1. Assuming a population size of 2,000,000 individuals less than 4 years old in Saudi Arabia (based on the 2022 Saudi census) [34], 95% confidence interval (CI), and a 50% expected frequency, a minimum sample size of 384 participants was determined. However, the final sample size comprised 391 participating parents.

The questionnaire consisted of three sections. The Section 1 collected demographic information, including the child’s age, gender, and nationality, as well as parental age and socioeconomic status. The Section 2 assessed parental and first-degree family history of food allergies. The Section 3 evaluated maternal awareness and practices regarding the timing of introducing potentially allergenic foods. The analysis specifically focused on the timing of allergenic food introduction directly into the infant’s or toddler’s diet (liquid, semi-solid, and solid forms). Exposure through breast milk was not analyzed, as previous studies have shown that dietary proteins (e.g., cow’s milk, egg, peanut, and wheat) are present in human milk only at very low concentrations and with substantial inter- and intra-individual variability [29,35].

Informed consent was obtained electronically from all participants prior to the commencement of the survey. The study was approved by the Institutional Review Board of Umm Al-Qura University (Approval No. HAPO-02-K-012-2023-10-1837). Participation was voluntary, and all responses were anonymized.

Data were analyzed using IBM SPSS Statistics software (version 23.0; IBM Corp., Armonk, NY, USA). Data were first screened for completeness and eligibility. Incomplete responses and questionnaires that did not meet the eligibility criteria were excluded prior to analysis. Categorical variables were summarized using frequencies and percentages. The primary outcome was the timing of the introduction of specific allergenic foods (egg, wheat, peanut, tree nuts, sesame, and seafood). Timing of food introduction was categorized into predefined age groups and dichotomized for selected analyses as early introduction (<12 months) versus delayed introduction (≥12 months or not introduced). Associations between categorical variables were assessed using the chi-square (χ^2^) test. Specifically, associations between the early introduction of allergenic foods and potential influencing factors, such as the presence of eczema, family history of food allergy, parental age, and parental education level, were evaluated. All statistical tests were two-tailed, and a *p*-value < 0.05 was considered statistically significant.

## 3. Results

### 3.1. Demographic Characteristics of the Study Participants

A total of 391 parents participated in the study. The children’s ages were distributed as follows: 97 (24.8%) were younger than 12 months, and 294 (75.2%) were aged 12–48 months. The detailed age distribution is presented in Table 1. More than half of the children were female (59.8%), while 40.2% were male. Among the parents, the majority were aged 30–40 years (44.8%). Nearly all parents were married (94.9%), whereas 3.6% were divorced and 1.5% were widowed. Approximately three-quarters of the participants held a bachelor’s degree or higher. In terms of maternal occupation, more than half of the mothers were housewives (53.5%), 42.5% were employed, and 4.1% were students (Table 1).

### 3.2. Prevalence of Parent Reported Food Allergy and Eczema

A total of 11.3% of children were reported to have food allergies by the parents. Among those with food allergies, 13.6% were self-diagnosed by parents, 40.9% were diagnosed by a healthcare provider, and 11.4% by an allergy specialist (Table 2). Additionally, 14.6% of children were reported to suffer from eczema. Of these, the diagnosis was most frequently established by general practice/primary care physicians (36.8%), followed by dermatologists (19.3%). Approximately one-third of participants (29.2%) reported a family history of food allergy (Table 3).

### 3.3. Timing of Allergenic Food Introduction

**Figure 1:**
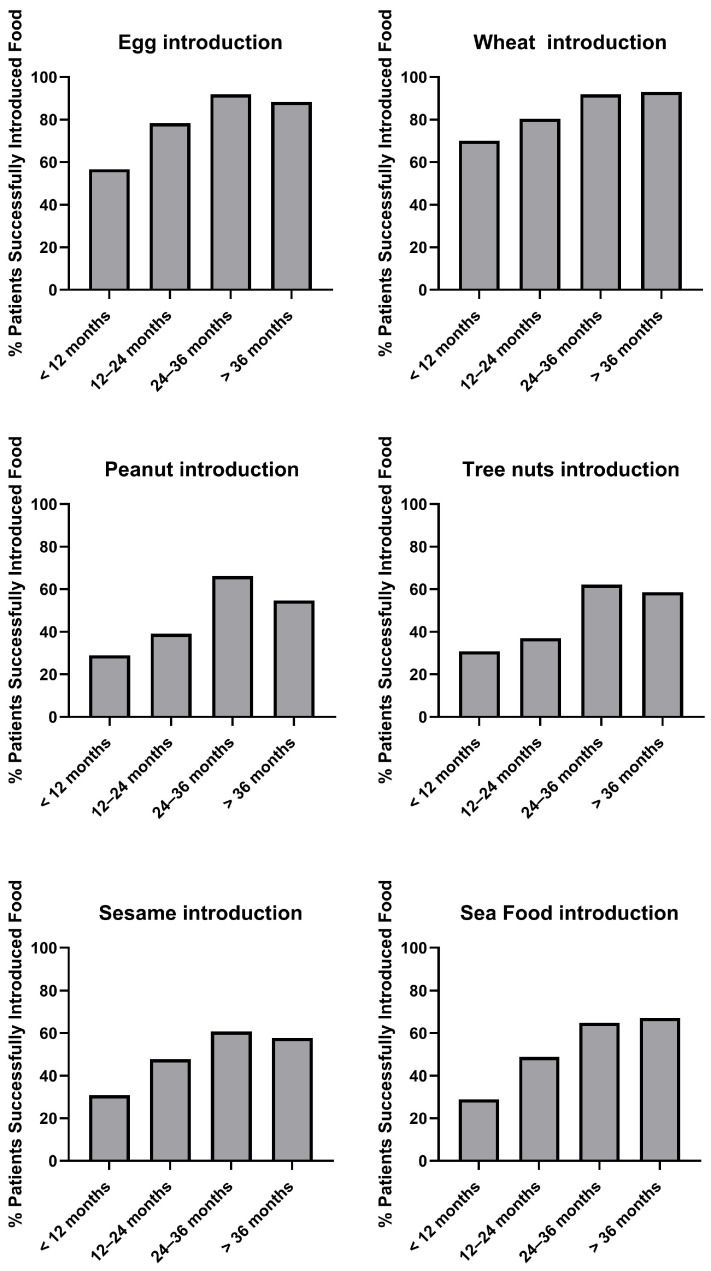
Timing of allergenic food introduction among study participants.

The rate of early introduction (<12 months) was notably higher for egg (43.3%) and wheat (71.1%) compared to other allergenic foods such as peanut (28.9%), tree nuts (30.9%), sesame (30.9%), and seafood (28.9%) (Figure 1). Across all food categories, the proportion of children introduced to allergenic foods increased progressively with age. Despite this upward trend, a significant proportion of children had not been introduced to several allergenic foods even beyond 36 months of age. Specifically, 11.7% of children had not been introduced to egg, 45.3% to peanut, 32.8% to seafood, 42.2% to sesame, and 41.4% to tree nuts (Figure 1). These findings reflect a clear distinction between commonly consumed staple foods—such as egg and wheat—and other foods perceived as high-risk allergens, including peanuts, nuts, sesame, and seafood.

### 3.4. Association Between Allergenic Food Introduction and History of Eczema or Family History of Food Allergy

Among the 391 children assessed, several differences were observed in the timing of allergenic food introduction between those with and without eczema. Children with eczema were significantly more likely to have egg introduced early compared to children without eczema (89.5% vs. 76.9%, *p* = 0.035; OR = 2.55, 95% CI 1.05–6.16). Introduction of tree nuts was also more common among children with eczema (59.6% vs. 45.2%, *p* = 0.046; OR = 1.79, 95% CI 1.01–3.17). Furthermore, seafood introduction showed a significant difference, with higher introduction rates in children with eczema (66.7% vs. 50.6%, *p* = 0.031; OR = 1.95, 95% CI 1.08–3.53). No statistically significant differences were observed between groups regarding the introduction of wheat, peanuts, or sesame (Table 4).

Next, the introduction of allergenic foods was compared between children with and without a family history of food allergies. Children with a family history of food allergy showed significantly higher rates of tree nut introduction compared to those without such history (55.3% vs. 44.0%, *p* = 0.043; OR = 1.57, 95% CI 1.01–2.43). Similarly, seafood introduction was significantly more frequent among children with a family history of food allergies (62.3% vs. 49.1%, *p* = 0.019; OR = 1.71, 95% CI 1.2–2.67) (Table 5).

### 3.5. Awareness of the Benefits of Early Introduction of Allergenic Foods

**Figure 2:**
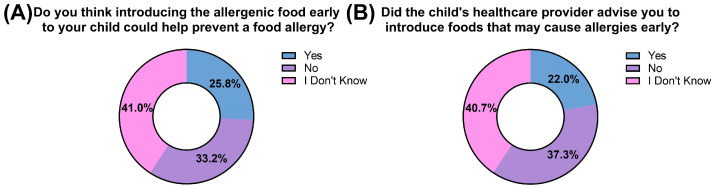
Awareness of the benefits of early introduction of allergenic foods.

Only a minority (25.8%) of participants were aware of the potential preventive effect of the early introduction of allergenic foods on food allergies. In contrast, 40.9% reported that they did not know whether early introduction could help prevent food allergies. Regarding healthcare provider guidance, 22.0% of respondents indicated that they had received advice to introduce allergenic foods early, while 37.3% reported not receiving such advice and 40.7% were unsure (Figure 2).

## 4. Discussion

The parent-reported prevalence of food allergy (11.3%) and eczema (14.6%) observed in this cohort reflects the global upward trend in atopic conditions among children [3,7,36]. These findings are also consistent with the limited but growing national evidence reported in Saudi Arabia [8,9]. These data emphasize an urgent need for preventive strategies that target early immune development in infancy. Early exposure to food antigens through the gastrointestinal tract favors the development of regulatory T cells and other immune mechanisms that suppress allergic responses [37,38]. This contrasts with sensitization through damaged skin (as in infants with eczema), which can promote Th2-dominant immunity and IgE production, increasing the risk of subsequent food allergy [39,40]. Importantly, this mechanistic rationale is supported by prospective randomized clinical trials: for example, the LEAP trial demonstrated that early introduction of peanut in high-risk infants substantially reduced the risk of peanut allergy, and the EAT trial suggested that early dietary introduction of multiple common allergens may reduce food allergy incidence in the general infant population [12,15,17]. Thus, optimizing early-life food exposures to support mucosal tolerance, while protecting skin-barrier integrity, may be central to strategies aiming to curb the rising burden of pediatric food allergy [41].

The timing of allergenic food introduction in our cohort demonstrated a clear pattern: commonly consumed foods such as egg and wheat were more likely to have been introduced by 36 months, whereas foods perceived as high-risk—such as peanuts, tree nuts, sesame, and seafood—were frequently delayed, with many children still unexposed after 36 months. This pattern aligns with international surveys reporting greater parental confidence with familiar foods and hesitancy toward introducing allergens perceived as potentially risky [42,43]. National survey data from the United States indicate that many caregivers continue to delay peanut and tree-nut introduction despite guideline changes [31]. A birth-cohort study from the United Kingdom similarly found that 21% of infants had not been introduced to egg and 35% had not been introduced to tree nuts by 12 months [44]. Japanese population data likewise show delayed introduction of fish, shellfish, peanuts, tree nuts, and fish eggs well into the second and third years of life [45]. Together, these findings suggest a global pattern of delayed introduction for foods perceived as high risk.

We also observed that infants and toddlers with eczema were more likely to have exposure of certain allergenic foods—specifically egg, tree nuts, and seafood. Similarly, children with a family history of food allergy were more likely to have exposure to tree nuts and seafood. These findings may reflect increased parental vigilance or earlier engagement with healthcare providers among families with prior experience of atopic disease. This is aligned with updated recommendations endorsing early introduction even in higher-risk infants [20,21]. Recent U.S. data similarly indicate increased counseling and earlier introduction among high-risk infants [23]. In contrast, the UK cohort reported greater avoidance rather than earlier exposure among such families [44]. These contrasting patterns may suggest that parental decision-making remains variable and may depend heavily on physician guidance and perceived safety rather than risk status alone.

Despite these findings, maternal awareness of the preventive benefits of early allergen introduction remained limited; only 25.8% of mothers recognized its potential role in reducing food allergy risk. Furthermore, just 22% reported receiving professional advice to introduce allergenic foods early. These findings are consistent with prior studies from the United States that have documented similar gaps in parental knowledge and counseling [23,31]. Together, these results highlight a substantial gap in counseling practices and suggest that emerging evidence supporting early introduction may not yet be fully integrated into routine pediatric care within the region.

Several factors may contribute to the limited awareness observed. Although the majority of parents in our cohort had high educational attainment, awareness of early allergen introduction remained low, indicating that formal education does not necessarily ensure familiarity with evolving allergy prevention recommendations. Prior studies examining maternal education and feeding practices have reported mixed or even contradictory associations [30,33], suggesting that feeding decisions are shaped by a complex interplay of factors rather than education level alone. In the Saudi context, cultural perceptions that certain foods—particularly nuts and seafood—are unsafe for infants may foster caution, especially when amplified by extended-family advice. Variability in pediatric guidance and limited access to allergy specialists in some regions likely also influence parental practices. The finding that only a minority of children with food allergy were diagnosed by specialists further highlights potential barriers to accessing allergists or suboptimal referral pathways within primary care. Additionally, reliance on social media for health information may contribute to inconsistent or inaccurate messaging [46].

These findings have important clinical implications. First, they highlight the need for targeted educational initiatives directed at both parents and healthcare providers, particularly primary care clinicians who are often the first point of contact. Second, reinforcing discussions on early allergen introduction within routine well-child visits may help standardize the guidance families receive. Third, culturally tailored educational materials—such as simplified guidance sheets, visual aids, or structured anticipatory counseling—may support families in safely introducing allergenic foods at appropriate ages. Finally, improving access to allergy specialists when clinically indicated—such as in infants with suspected food allergy, severe eczema, or families requiring additional support—may facilitate accurate diagnosis and provide tailored preventive guidance.

This study has several strengths, including the inclusion of a broad age range, and allowing for the assessment of real-world practices across early childhood. However, some limitations should be noted. The cross-sectional design precludes temporal or causal inference. Parental recall may introduce bias, particularly for feeding practices occurring months or years prior. Allergic conditions were based on parent-reported diagnoses, which may lead to misclassification or under/overestimation, especially in regions with limited access to specialist evaluation. The modest overall sample size may limit the precision of subgroup analyses. Additionally, recruitment through social media platforms may introduce selection bias; nevertheless, an official government report indicates that more than 95% of the Saudi population aged 20–59 years actively uses social media applications, supporting the accessibility of this recruitment method [46]. Furthermore, this study did not account for exposure to food allergens through breast milk, which can vary depending on maternal diet, timing, and quantity of allergen intake.

Future research should incorporate clinical verification of food allergy and adopt longitudinal designs to more precisely capture timing, frequency, and consistency of allergen introduction. Qualitative research exploring parental beliefs, cultural influences, and barriers to guideline adherence may offer deeper insight. Educational interventions delivered through primary care or digital platforms should also be evaluated for their potential to improve adherence to early-introduction recommendations in the region

## 5. Conclusions

Maternal awareness of early allergen introduction remains low in the Makkah region, and delayed introduction of several high-risk foods such as nuts, seafoods and sesame was common. Children with eczema or a family history of allergy exhibited higher cumulative introduction of some allergens, suggesting that prior experience with atopic disease may influence feeding decisions. These findings highlight the need for standardized counseling in primary care, culturally appropriate educational materials, and timely referral to allergy specialists. Strengthening these practices may reduce unnecessary delays and support evidence-based food-allergy prevention strategies in Saudi Arabia.

## Figures and Tables

**Table 1:** Demographic characteristics of the study participants (N = 391).

Characteristic		Frequency	Percentage
Age	<12 months	97	24.8
	12–24 months	92	23.5
	24–36 months	74	18.9
	>36 months	128	32.7
gender	Female	234	59.8
	Male	157	40.2
Parent’s Age	Less than 30 years	158	40.4
	30–40 years	175	44.8
	More than 40 years	57	14.6
Marital Status	Married	371	94.9
	Divorced	14	3.6
	Widow	6	1.5
Father’s Educational Level	High school level or lower	101	25.8
	Bachelor’s degree	230	58.8
	Master, PhD or equivalent	60	15.3
Mother’s Educational Level	High school level or lower	88	22.5
	Bachelor’s degree	263	67.3
	Master, PhD or equivalent	40	10.2
Mother’s Occupation	Student	16	4.1
	Unemployed (housewife)	209	53.5
	Employed	166	42.5
Number of children	1	141	36.1
	2	92	23.5
	3	68	17.4
	More than 3	90	23.0

**Table 2:** Prevalence of parent reported food allergy and eczema (N = 391).

Characteristic		Frequency	Percentage
Parents reported food allergy	No	347	88.7
	Yes	44	11.3
If yes, diagnosis made by	Self-diagnosis	6	13.6
	Healthcare provider	18	40.9
	Allergy specialist	5	11.4
Parents reported eczema	No	334	85.4
	Yes	57	14.6
If yes, diagnosis made by	Self-diagnosis	6	10.5
	Healthcare provider	21	36.8
	Dermatologist	11	19.3

**Table 3:** Family member with reported food allergy.

Characteristic	Frequency	Percentage ^1^
Father	43	11
Mother	44	11.3
Siblings	46	11.8
No affected family member	277	70.8

^1^ Percentages may exceed 100% because the categories are not mutually exclusive; a child may have more than one family member (e.g., father and sibling) with a history of allergy.

**Table 4:** Introduction of allergenic foods among children with eczema versus those without eczema.

Characteristic		Children Without Eczema (N = 334)	Children with Eczema (N = 57)	*p*-Value	Odd Ratio (95% Confidence Interval)
Egg introduction	Yes	257 (76.9%)	51 (89.5%)	0.035	2.55 (1.05–6.16)
	No	77 (23.1%)	6 (10.5%)		
Wheat introduction	Yes	279 (83.5%)	51 (89.5%)	0.325	1.68 (0.69–4.1)
	No	55 (16.5%)	6 (10.5%)		
Peanut introduction	Yes	154 (46.1%)	29 (50.9%)	0.566	1.21 (0.69–2.12)
	No	180 (53.9%)	28 (49.1%)		
Tree Nuts introduction	Yes	151 (45.2%)	34 (59.6%)	0.046	1.79 (1.01–3.17)
	No	183 (54.8%)	23 (40.4%)		
Sea Food introduction	Yes	169 (50.6%)	38 (66.7%)	0.031	1.95 (1.08–3.53)
	No	165 (49.4%)	19 (33.3%)		
Sesame introduction	Yes	162 (48.5%)	31 (54.4%)	0.474	1.27 (0.72–2.22)
	No	172 (51.5%)	26 (45.6%)		

**Table 5:** Introduction of allergenic foods among children with and without a family history of food allergies.

Characteristic		Children Without Family History of FA (N = 227)	Children with Family History of FA (N = 114)	*p*-Value	Odd Ratio (95% Confidence Interval)
Egg introduction	Yes	218 (78.7%)	90 (78.9%)	0.957	1.01 (0.59–1.73)
	No	59 (21.3%)	24 (21.1%)		
Wheat introduction	Yes	234 (84.5%)	96 (84.2%)	0.947	0.98 (0.54–1.79)
	No	43 (15.5%)	18 (15.8%)		
Peanut introduction	Yes	125 (45.1%)	58 (50.9%)	0.317	1.26 (0.81–1.95)
	No	152 (54.9%)	56 (49.1%)		
Tree Nuts introduction	Yes	122 (44.0%)	63 (55.3%)	0.043	1.57 (1.01–2.43)
	No	155 (56.0%)	51 (44.7%)		
Sea Food introduction	Yes	136 (49.1%)	71 (62.3%)	0.019	1.71 (1.2–2.67)
	No	141 (50.9%)	43 (37.7%)		
Sesame introduction	Yes	138 (49.8%)	55 (48.2%)	0.824	0.94 (0.61–1.45)
	No	139 (50.2%)	59 (51.8%)		

## Data Availability

The raw data supporting the conclusions of this article will be made available by the authors upon reasonable request.
